# A precision approach to translational research in acute lung injury

**DOI:** 10.1093/ajrccm/aamag143

**Published:** 2026-03-27

**Authors:** Guillermo M Albaiceta, Claudia C dos Santos, Rachel L Zemans, Wolfgang M Kuebler

**Affiliations:** Servicio de Medicina Intensiva, Hospital Universitario Central de Asturias, Oviedo, Spain; Departamento de Biología Funcional, Instituto Universitario de Oncología del Principado de Asturias, Universidad de Oviedo, Oviedo, Spain; Instituto de Investigación Sanitaria del Principado de Asturias, Oviedo, Spain; Centro de Investigación Biomédica en Red (CIBER)-Enfermedades respiratorias, Madrid, Spain; Interdepartmental Division of Critical Care, University of Toronto, Toronto, Ontario, Canada; Keenan Research Centre for Biomedical Science, Unity Health Toronto, Toronto, Ontario, Canada; Division of Pulmonary and Critical Care Medicine, Department of Internal Medicine, University of Michigan, Ann Arbor, MI, United States; Cellular and Molecular Biology Program, University of Michigan, Ann Arbor, MI, United States; Institute of Physiology, Charité-Universitätsmedizin Berlin, Corporate Member of the Freie Universität Berlin and Humboldt Universität zu Berlin, Berlin, Germany; DZL (German Centre for Lung Research), partner site Berlin, Berlin, Germany; DZHK (German Centre for Cardiovascular Research), partner site Berlin, Berlin, Germany; Departments of Surgery and Physiology, University of Toronto, Toronto, Ontario, Canada

Translational research aims to develop and test hypotheses that go beyond the identification of pathogenetic mechanisms to promote the development of new knowledge and therapeutic strategies that can be applied at the bedside.^1^ However, this approach has struggled to show significant advances in critical care medicine. Intensive care medicine has decreased mortality rates using strategies that decrease iatrogenic injury but not by modifying the natural history of the diseases or syndromes that require critical care.

The field of acute lung injury (ALI) provides one of the best examples of these difficulties. The study of the distribution of mechanical stress within the lung and the identification of ventilator-induced lung injury in animal models led to the development of protective ventilatory strategies, which ultimately were proven effective in clinical trials. Cumulative findings from preclinical and clinical research were incorporated into guidelines and improved the outcome of patients with acute respiratory distress syndrome (ARDS). Other translational approaches have, however, failed to yield similar improvements. This is of special importance in drug development, where more than 90% of compounds that appear safe and effective in animals fail during human clinical trials and never reach the patient.^2^ Specifically, in ARDS, not a single pharmaceutical intervention developed and validated in the lab, including gene and cell therapies, has so far significantly improved outcomes. Therefore, a reappraisal of the translational approach is warranted.

The objective of this perspective is to review the limitations of the current approach to translational critical care (mainly based on animal models) and the novel alternatives emerging from preclinical technologies, clinical data, and in silico approaches to improve precision in critical care. Precision medicine seeks to integrate the mechanistic understanding of disease with individual variability in biology, physiology, and clinical context to identify patient-specific therapies. Rather than attempting to replace animal studies outright, computational, in vitro, and microphysiological models should be integrated with limited in vivo and human data to build convergent evidence (Figure 1). This way, replacement of animals is prioritized, and the strengths of each experimental model can help address the concerns raised by the limitations of the others.

**Figure 1:**
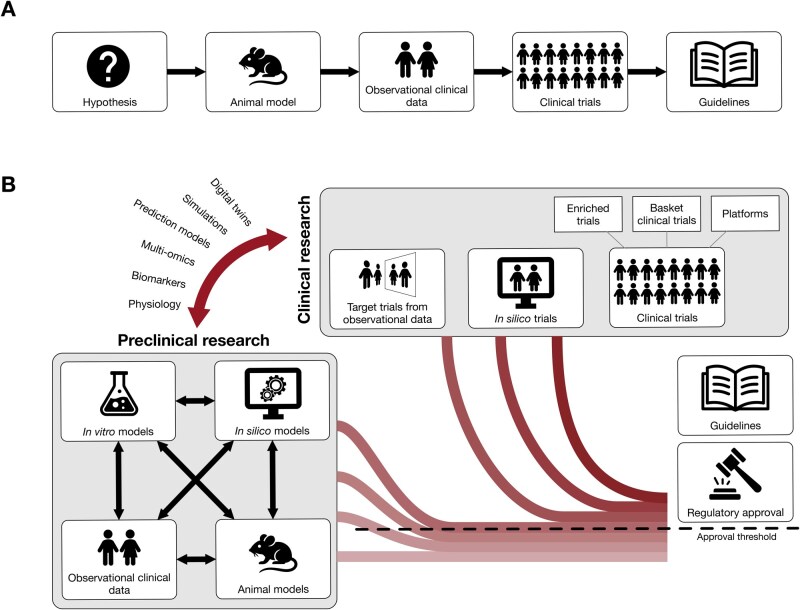
Pathways of translational research. (A) Classic translational research, with a linear pathway from the hypothesis to the clinical application of the findings. (B) An alternative approach to translational research in which there is a crosstalk among preclinical models, using in silico, in vitro, and in vivo approaches, that links and informs clinical research. Integrating artificial intelligence, mechanistic simulation, and digital twinning into preclinical drug discovery for acute lung injury offers a path to balance precision with pragmatism. By anchoring models in real-world human datasets (multiomics, imaging, ventilatory waveforms, bedside physiology, etc.), predictive features and patient-specific treatable traits can be defined. These predictions can then be validated in scalable preclinical platforms, such as lung-on-chip systems or precision-cut lung slices, ensuring biological fidelity while limiting costly iterations. Small, biomarker-driven human experimental studies, guided by digital twin-informed simulations, can confirm target engagement and short-term mechanistic effects, reducing the risk of failure in larger pragmatic, biomarker-enriched trial designs.

## Limitations of experimental models

Our continued failure to translate successful pharmacological interventions from the lab into the clinical realms has highlighted the limitations of existing models and generated significant skepticism about the translational relevance of experimental models.^3^ Some of these limitations are model inherent, while others relate to the way in which we design preclinical studies and experimental protocols and analyze and report data. Table 1 summarizes these limitations and how they may potentially be overcome. Among these, some are important to highlight.

**Table 1:** Limitations of animal models in modeling ARDS.

Problem	Specification	Solution
**Model inherent**		
**Species inherent**	Differences in biomechanics, hemodynamics, immune responses, and genetic traits	Large animal models, humanized mice
**Untrained immune system/lack of diversity of microbiome**	Use of specific-pathogen free-mice with untrained immune system	Use of humanized or wildling mice; control for microbiome
**Study design**		
**Standardization**	Lack of diversity in terms of genetic traits, age, sex, microbiome, or disease triggers	Introducing diversity into animal models
**Age**	Common use of young animals	Animal models should mimic the appropriate human age group affected
**Comorbidities**	Lack of comorbidities	Introducing comorbidities into animal models
**Randomization**	Variable blinding and randomization strategies	Appropriate blinding and randomization and reporting thereof as in clinical trials
**Controls**	Variable controls for therapies (none, vehicle, placebo)	Standardization of controls
**Outcomes**	Precursors, easy-to-measure, disease surrogates	Clinically relevant outcomes
**Experimental group size**	Small group sizes with inadequate power, required sample size often not predetermined	Precalculated group size based on primary outcome parameters and adequate power
**Multicentric trials**	Preclinical trials are usually monocentric	Multicentric preclinical trials
**Experimental protocols**		
**Experimental time course**	Short-term experiments that do not correspond to disease course in humans	Models > 72 h
**Disease triggers**	Sudden onset of disease that does not correspond to latency in humans	Human disease triggers (eg, bacterial infection rather than LPS)
**Drug dosing schedules and regimen**	Testing of scenarios of uncertain clinical relevance (eg, prophylactic interventions)	Testing of clinically relevant scenarios
**Positioning**	(Unphysiological) supine positioning	Species-appropriate positioning where possible
**Temperature**	Frequent lack of temperature control	Appropriate temperature control
**Ventilation**	Animals breathing spontaneously with no monitoring of respiratory rate or oxygenation status Mechanical ventilation without sighs, recruitment maneuvers, or suctioning	Monitor respiration and oxygenation Mechanical ventilation should mimic clinical ventilation strategies
**ICU support**	Fluid support, antibiotics, or catecholamines used in the human ICU setting are not routinely applied	Support measures should mimic relevant clinical scenarios, in particular for drug testing
**Analysis and reporting**		
**ARDS criteria**	Clinical criteria of ARDS not assessed, reported, or met	Rigorous assessment and reporting, in particular for drug testing
**Endpoints**	Use of surrogate endpoints with questionable clinical relevance Use of relative endpoints that provide little information on disease severity	Use of clinically relevant endpoints Use of absolute endpoints such as wet/dry weight ratio
**Loss to follow-up**	Animal loss not rigorously reported, survival bias	Follow intention-to-treat principle, rigorous reporting for all animals entering the study
**Laboratory techniques**	Nuances neither recognized nor reported	Detailed reporting, multicentric trials
**Statistics**	Simplistic statistical analysis, lack of accounting for potential confounders	Adequate statistical analyses

Abbreviation: ARDS, acute respiratory distress syndrome; LPS, lipopolysaccharide.

### Animals are not humans

Animals and patients differ in key traits relevant to the pathophysiology of ALI, including biomechanics and hemodynamics, their response to injury, and their immune system responses.^4^ Small animals are typically kept under specific pathogen-free conditions with untrained immune systems, with a common microbiome. Some human traits may be reproduced experimentally (eg, by use of large animals, such as pigs, or humanized mice bearing a human immune system).^5^ Even an alternative approach such as the zebrafish model may mimic inflammatory responses to pathogens or toxins and could be useful for drug testing.^6^

### Study design

In animal models, disease is typically triggered in genetically identical, young, otherwise healthy animals by defined noxious or pathogenic agents at distinct time points. This contrasts to patients in which previous medical history, exposures, and genetic factors are diverse and typically unknown. Of special importance, sex differences, often neglected in basic research, must be considered in both preclinical and clinical studies. There is ample evidence for a critical role of sex as biological variable in acute lung injury and ARDS, both in animal models^7^ and clinical scenarios with significantly higher mortalities in males than females.^8^ As such, sex-inclusive research should be the standard for scientific rigor and excellence in biomedical research.

Preclinical animal studies also often lack rigorous a priori randomization and blinding strategies, use a variety of treatment controls (none, vehicle, or placebo) and small group sizes with insufficient power, fail to consider intention to treat or loss to follow-up, and are not performed as multicentric trials, all limiting rigor and reproducibility. A recent meta-analysis^9^ of animal-to-human translation found that approximately 50% of therapies progressed from animal studies to initial human studies, 40% advanced to human randomized clinical trials (RCTs), and only 5% ultimately achieved regulatory approval. The findings from the meta-analysis were consistent with an 86% concordance between positive results in animal studies and subsequent clinical trials. Together, these data suggest that although bench-to-bedside translation remains limited, robust study designs such as RCTs in animal models may reinforce generalizability and improve the translation of new therapies to the bedside.

### Experimental protocols

Setting and time course of animal models typically differ considerably from the clinical scenario.^10^ Most animal models are acute (hours rather than days) in onset and follow-up, which can affect therapeutic responsiveness and clinical relevance of endpoints (please also see Timing in experimental models and patients below for further discussion). Similarly, time points of drug dosing (often prophylactic rather than therapeutic) do not correspond to clinically relevant scenarios and are often not controlled for circadian rhythm. Animal positioning, temperature control, ventilation regimes, or ICU support frequently fail to replicate meaningful clinical scenarios.

### Physiological approach to animal models

While animal models allow for in-depth mechanistic analyses (using multiomics approaches or genetically engineered organisms), many studies do not meet or report the characteristic criteria of clinical ARDS, thus limiting their translational relevance. Among approximately 3000 articles on ALI in animal models published between 2000 and 2020, less than one-quarter reported data on physiological dysfunction (ie, impaired oxygenation, respiratory distress, or impaired lung mechanics).^11^ Use of clinically relevant and absolute endpoints providing reproducible information on disease severity becomes critically relevant when advancing from basic mechanistic studies to preclinical and clinical drug testing.^12^

### Lack of systematic reviews

In clinical research, systematic reviews provide invaluable information on clinical associations and therapeutic effectiveness. Analogously, systematic reviews in preclinical research may help discriminate between open vs already answered questions, thereby contributing to the reduction of animal experiments according to the 3R (reduction and replacement of animals and refinement of procedures) principle, increase the precision of estimated treatment effects used in calculating the power for subsequent human trials, and evaluate the translational value (or lack thereof) of animal models. At present, however, systematic reviews in preclinical research are rare and often limited to the analysis of models and how they match clinical scenarios. The effectiveness of therapeutic interventions in preclinical scenarios is less commonly assessed, largely because of (1) difficulties to compare different experimental study designs, species, drug dosing, and so on; (2) limited scientific incentive for reproducing preclinical drug studies; (3) the economic necessity to bring novel therapies into clinical trials as fast as possible; and (4) systematic underreporting of negative findings in preclinical research, introducing inherent bias. To overcome these limitations would require systematic registration and reporting of all preclinical drug trials and widespread recognition of (and funding for) the necessity of experimental validation by independent groups.

Ultimately all models have limitations. In the words of British statistician George Box, “all models are wrong, but some are useful.”^13^ The extent to which experimental models may yield important information critically depends on the question asked. Yet, the more the study is geared toward clinical translation, the more critical it becomes to carefully model all aspects of the clinical disease.

## Alternatives to animal models

**Figure 2:**
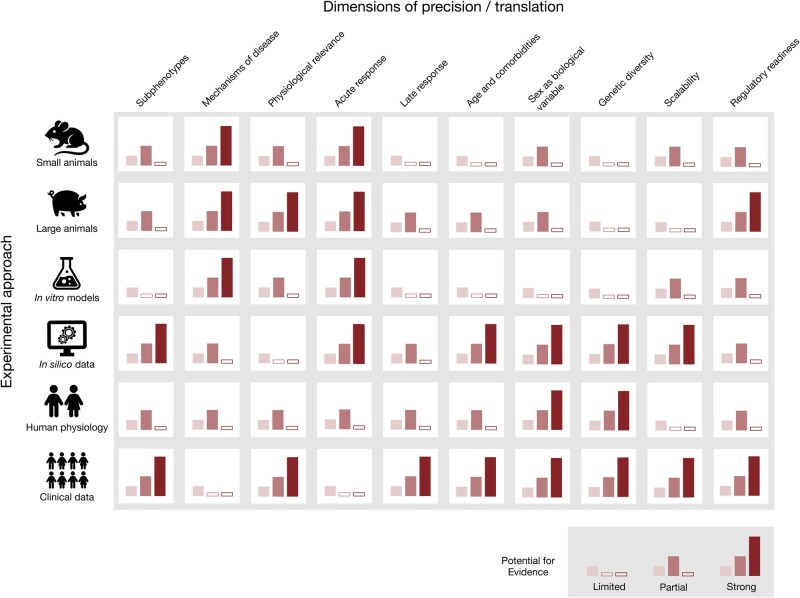
Strengths and weaknesses of different models used for translational research. The figure summarizes the relative strengths and limitations of commonly used translational approaches across key dimensions, including biological heterogeneity, temporal disease evolution, and clinical realism. While no single strategy captures all dimensions, integration of experimental, computational, and human-based approaches enables a precision-oriented translational pipeline.

Physiology has always been key and often considered irreplaceable in critical care medicine. Despite this, *new approach methodologies* (NAMs), a collective term for approaches involving computational modeling, artificial intelligence (AI)–driven predictors, tests in cells, and organ-on-a-chip technologies, emerge as a complementary tool that allows researchers to add layers of evidence to their findings. Each of these models has its own set of strengths and weakness, summarized in Figure 2 and discussed in the following sections of this perspective.

In ARDS, Huh  et al.^14^ and Stucki  et al.^15^ demonstrated that a micro-engineered lung-on-chip can reproduce breathing mechanics and inflammatory responses. While such systems present considerable advances, they typically replicate specific aspects of the physiological scenario but fall short of adequately accounting for the combination of biomechanical (hemodynamics, ventilation), biochemical (circulating, multi-organ–derived mediators and metabolites), and immunological contexts.^16^ As an alternative, precision cut lung slices and isolated perfused human lungs overcome some of these limitations with respect to, for example, anatomical context and multicellularity. The extent to which they surpass animal models in the identification and testing of pharmacological targets remains, however, to be shown.

Computational (in silico) modeling is now one of the most rapidly advancing nonanimal translational tools in critical care medicine. While not ready to replace animal models, in silico systems are already replacing parts of the mechanistic and hypothesis-testing pipeline that used to depend on animals. Pioneering work by Hickling,^17^ 27 years ago, identified in a simple computer model the impact of recruitment in lung mechanics. More complex models aim to predict the development of ventilator-induced lung injury.^18^ These models can be applied to simulate “digital twins”^19^ and in silico clinical trials,^20^^,^^21^ which may help predict the effects of specific therapies.

As we move into the future, NAM-based research will need to become explicitly structured to validate and qualify microphysiological systems so that their data can be reliably used in drug development and regulatory decisions. The major limitation for moving to NAM-only first-in-human is that these approaches may not be ready for prime time. First-in-human trials continue to require robust safety data (often from animal models) and/or regulatory precedent. Importantly, NAMs are still gaining validation and regulatory acceptance. Critical-care syndromes (like ARDS, multi‐organ failure, shock) are highly complex, systemic, and variable; replicating that complexity with NAMs alone remains a significant challenge, since we are still far from understanding the underlying pathophysiologies and, hence, what needs to be included in the models. Regulators still require animal safety/toxicity data or human safety cohorts before efficacy trials, making full replacement of animal models rare to date. It’s likely that future first-in-human studies will increasingly incorporate NAM-derived data (eg, for mechanism, biomarker identification, dosing rationale) alongside or replacing some animal data. For critical-care applications, a hybrid approach is probable: NAMs combined with limited animal work and human observational/monitoring data, followed by human trials.

More recently, AI has been used to improve how we analyze, interpret, and *translate* data from animal models to human biology. Artificial intelligence and machine-learning is being used to standardize and automate histological and imaging analyses in animal models of lung injury,^22^ reducing observer variability and enhancing quantification of injury markers such as neutrophilic infiltration, which improves internal validity and model comparability across studies. Data-driven tools further help integrate high-dimensional omics data (eg, transcriptomics, multiomics, spatial transcriptomics) from preclinical ALI models with human datasets, facilitating the identification of shared molecular signatures/pathways that bridge species differences. Artificial intelligence–assisted multiscale biomechanical modeling is being used to estimate tissue stresses and biomechanical changes that traditional measures miss,^23^ enabling a deeper understanding of injury progression in animal lungs that may better reflect human pathophysiology, thus improving phenotypic fidelity, quantitative rigor, and data integration; reducing noise; improving reproducibility; and enabling more robust cross-species comparisons.

## Syndromes, not diseases

From the interaction between the triggering etiology and host factors emerges the syndromes diagnosed in critically ill patients. These syndromes may be split in subphenotypes based on identifiable properties, such as biomarkers or clinical or physiological variables. Phenotypes and subphenotypes that share a specific underlying biological mechanism are known as endotypes.^24^ All these variants may require specific treatments.^25^ Capturing this individual variability in uniform models of translational research is a significant challenge.

The concept of treatable traits acknowledges the heterogeneity of distinct biological and clinical syndromes and their manifestation in individual patients. Key modifiable traits may include abnormalities in respiratory mechanics (such as poor compliance or variable recruitability), gas exchange derangements (severe hypoxemia or hypercapnia), and distinct inflammatory phenotypes (hyperinflammatory vs hypo-inflammatory), as well as other factors such as right ventricular dysfunction, infection, fluid overload, and coagulopathy, each guiding specific, and possibly different, targeted interventions.

One of the reasons behind the failure in translation of most of the findings coming from basic research is the diversity of patients’ responses to a given condition. Recent studies have proposed the existence of subphenotypes in standardized animal models,^26^ which may be dependent on the initial insult and the genetic background. Addition of proinflammatory agents, such as lipopolysaccharide, drives the phenotype toward a hyperinflammatory state. The genetic background results in differences in the expression of genes involved in apoptosis or antioxidant response, which influence the subphenotype in a model of lung injury caused by bleomycin instillation.^27^ Alternatively, or in parallel, subphenotypes may simply emerge randomly in a complex system along different trajectories of possible outcomes.

The extent to which these variants correspond to clinical phenotypes and predict disease trajectories and drug responses in humans remains to be shown. To improve translation, the results of preclinical research should consider different subphenotypes to mimic patient scenarios. A precision-based approach to critical care syndromes must identify which experimental models best resemble the underlying pathophysiology to facilitate precision in preclinical drug testing. Rather than syndromes, actionable mechanisms shared by experimental models and patients should drive this translation. This implies the discovery of common, specific biomarkers, that could help establish these phenotypic correspondences between models and patients.

## Timing in experimental models and patients

Another major issue that may limit the translation of research findings to clinical practice is the timing of ALI. Experimental models are developed in a strict time frame, where the moment of injury is known, and the pathophysiology is almost immediately evident. In contrast, patients with lung damage are usually admitted to the critical care unit once the disease has evolved, often days to weeks after the initial insult.^28^ In the context of delayed presentation, the prevalent pathophysiology may be dramatically different from the early phase of the disease commonly simulated in preclinical models.

In ALI, there is ample evidence that lung repair mechanisms start early after injury. Early inflammatory responses may cause severe damage due to the release of cytokines and chemokines, proteases, reactive oxygen species, and other molecules that disrupt the alveolar architecture. At the same time, collagen deposition may be observed few hours after damage.^29^ The long-term success of early therapeutics may reflect control of early noxious inflammation. However, disruption of these responses later in the course of illness may be harmful, as they are required for repair. The role of neutrophils in injury and repair is representative of this time-dependent functional duality. Neutrophil depletion early in the course of experimental lung damage, or even before injury is induced, prevents tissue disruption and preserves gas exchange in models of ventilator-induced lung injury, lipopolysaccharide (LPS) administration, or transfusion-related ALI;^30^ in contrast, neutrophil depletion in established injury delays repair as it impairs tissue remodeling.^31-33^

Transmigration of neutrophils across the alveolar epithelium activates its proliferation and repair.^34^ Once in the alveoli, neutrophils clear debris and release proteases that deposit collagen, providing a scaffold for epithelial regeneration, counteracting an excessive accumulation of extracellular matrix that could lead to fibrosis. Therefore, blockade of neutrophil functions or depletion at this timepoint can cause abnormal tissue repair. There is evidence that other inflammatory molecules, such as interleukins-1^35^ and -6^36^ are also involved in epithelial repair, which may explain the contradictory results in clinical trials targeting these molecules.^37^^,^^38^ Similarly, mesenchymal stromal cells may be beneficial early, but detrimental late in the disease process.^39^

How then to translate findings from short-term models to the real-world patient? One option is to measure markers of repair in acute phase studies, for example, in studies testing an anti-inflammatory drug. Another option is developing animal models that can be maintained for longer periods of time. While prolonged (>48 h) large animal experiments are cost- and labor-intensive, they bear considerable potential for bridging the translational gap from bench to bedside.^40^ An effort to elucidate the repair mechanisms of the injured lung must be embraced, even with the concern that there may be only a small margin for improvement of processes that have already been optimized by evolutionary pressures.^41^

## Refining precision up to the cell level

The emergence of single-cell technologies that allow characterization of specific responses at the level of specific cells may reveal novel mechanisms of injury and repair, particularly linked to specific cell populations. Traditionally (as estimated by histology), the lung was thought to contain approximately 40-50 distinct cell types. Recent single-cell RNA sequencing studies now identify more than 80-100 transcriptionally distinct cell populations.^42^ The abundance, function, and interaction of these distinct cells are dynamically altered over time by injury, repair, and treatment.^43^ Results from single-cell sequencing studies reveal that a substantial portion of the intracellular mechanisms activated by lung injury are cell specific. For instance, data on COVID-19 shows that the expression of proposed therapeutic targets such as interferon-γ or TGFβ is different in epithelial and inflammatory cells.^44^^,^^45^ Experimental models that include both aspects of injury and repair should ideally test cell-specific responses to therapy,^46^ but realistically modeling all these different cell types in NAM models may not be feasible. Bulk tissue analysis and systemic therapies that target a specific pathway may have the desired effect in a cell line in which that pathway is pathogenetic but may have off-target effects in other cell populations. Importantly, the therapeutic index of a specific strategy may be limited by its toxicity in alternate cell types. To be effective, novel interventions must aim to target not only a specific molecule/pathway but also a specific cell. Biotechnology approaches using functionalized nanoparticles or cell-specific vectors can deploy therapeutics to desired cellular and molecular targets. These strategies are now under development^47^^,^^48^ and may constitute a powerful tool for “double-precision” medicine.

## The translational potential of clinical data

Recognizing the translational gap between animal models and human critical care, there is growing interest in deploying humans themselves (in carefully designed studies) as the ultimate preclinical model, enabling direct investigation of disease processes, biomarker responses, and therapeutic modulation in a human-relevant context.^49^ While ethical and logistical constraints limit the extent to which disease can be induced or manipulated in volunteers/patients, human models offer biologically valid insight into mechanisms, dose response, and target specificity and thus can complement or even precede traditional animal studies. There are examples of this with healthy human endotoxin studies to model ALI.^50^ There may also be a tentative interest in exploring the use of N-of-1 studies in critical care to bridge the gap between population-level evidence and individualized care.

Finally, the expanding availability of large patient datasets and electronic health care records from clinical cohorts, national registries, and international data networks increasingly facilitates a reverse approach by unbiased screening or targeted testing of therapies that were prescribed for different indications but retrospectively demonstrate a clinical benefit in critical care scenarios. Such real-world analyses have been successfully applied to identify the association of specific treatments with favorable outcomes (such as tricyclic antidepressants with a reduced risk for mechanical ventilation and death in patients hospitalized for community-acquired pneumonia or COVID-19^51^) or to assess the performance of biomarkers, their incremental clinical utility and cost-effectiveness, or their potential to identify treatable traits, such as C-reactive protein to select patients with pneumonia who benefit from steroids.^52^ Precision-based translational research must identify the right treatment to the right patient, at the right time, and in the right cell.

## Conclusions

How can translational scientists balance precision with pragmatism for novel treatments for ARDS? The short answer is to build a tiered, measurable translational pipeline that identifies human-relevant targets and biomarkers early, tests mechanistic hypotheses in scalable ex vivo/high-throughput platforms and refined animal models, and then validates target engagement and short-term biologic effect in small, tightly phenotyped human experimental studies or adaptive early phase trials—using pragmatic clinical outcomes only once there is consistent mechanistic and safety evidence. In this pipeline, focused animal studies are integrated with other sources of data to build solid evidence.

Collaborative, interdisciplinary work and transparent data sharing—ideally through open, Findable, Accessible, Interoperable, and Reusable repositories—enhance model credibility and reproducibility, ensuring NAM-based research contributes meaningfully to translational and clinical progress. From the classical linear pathway from discovery to clinical application, translational research must move toward a multidimensional approach (Figure 1) in which, resembling Feyerabend’s proposal that “anything goes,”^53^ researchers and clinicians exploit multiple strategies, data sources, models, and preclinical results to build solid evidence aimed at specific cellular and molecular targets and patient population.

## Supplementary Material

aamag143_Supplementary_Data
